# Ultrasound-based deep learning model as an assistant improves the diagnosis of ovarian tumors: a multicenter study

**DOI:** 10.1186/s13244-025-02112-4

**Published:** 2025-10-16

**Authors:** Yanli Wang, Jiansong Zhang, Yifang He, Xiali Wang, Xiuming Wu, Weina Zhang, Min Gong, Dan Gao, Shunlan Liu, Peizhong Liu, Ping Li, Linlin Shen, Guorong Lyu

**Affiliations:** 1 Department of Ultrasound, The Second Affiliated Hospital of Fujian Medical University, Quanzhou, China; 2 School of Artificial Intelligence, Shenzhen University, Shenzhen, China; 3 Department of Clinical Medicine, Quanzhou Medical College, Quanzhou, China; 4 Department of Ultrasound, Quanzhou First Hospital, Quanzhou, China; 5 Department of Ultrasound, Zhangzhou Hospital, Zhangzhou, China; 6 Department of Ultrasound, Chengdu Third People’s Hospital, Chengdu, China; 7 Department of Ultrasound, The First Affiliated Hospital of Dalian Medical University, Dalian, China; 8 College of Engineering, Huaqiao University, Quanzhou, China; 9 Department of Gynecology and Obstetrics, Quanzhou First Hospital, Quanzhou, China

**Keywords:** Ovarian tumors, Deep learning, Diagnosis, Ultrasound

## Abstract

**Background:**

Deep learning (DL) models based on ultrasound (US) images can enhance the ability of radiologists to diagnose ovarian tumors.

**Materials and methods:**

This retrospective study included 916 women with ovarian tumors in southeast China who underwent surgery with clear pathology and preoperative US examination. The data set was divided into a training (80%) and a validation (20%) set. The test set consisted of 81 women with ovarian tumors from southwest and northeast China. DL models based on three backbone architectures, ResNet-50 (residual CNN), VGG16 (plain CNN), and Vision Transformer (ViT), were trained to classify benign, borderline, and malignant ovarian tumors. The diagnostic efficiency of primary US doctors combined with the DL model was compared with the ADNEX model and a US expert. Additionally, we compared the diagnostic performance of primary US doctors before and after being assisted by the integrated framework combining visual DL models and large language models.

**Results:**

(1) The accuracy of the ResNet50-based DL model for benign, malignant, and borderline ovarian tumors was 91.8%, 84.61%, and 82.60% for the test sets, respectively. (2) After visual and linguistic DL assistance, the accuracy of primary US doctors all improved in the test set (doctor A: 76.62% to 90.90%, doctor B: 76.62% to 90.90%, doctor C: 79.22% to 94.54%, doctor D: 76.62% to 95.95%, doctor E: 76.60% to 95.95%, respectively). (3) The diagnostic consistency of primary US doctors for validation and test sets also increased (doctor A: 0.671 to 0.912, doctor B: 0.762 to 0.916, doctor C: 0.412 to 0.629, doctor D: 0.588 to 0.701, doctor E: 0.528 to 0.710, respectively).

**Conclusions:**

A DL system combining an image-based model (vision model) and a language model was developed to assist radiologists in classifying ovarian tumors in US images and enhance diagnostic efficacy.

**Critical relevance statement:**

The established model can assist primary US doctors in preoperative diagnosis and improve the early detection and timely treatment of ovarian tumors.

**Key Points:**

- An ultrasound-based deep learning (DL) model was developed for ovarian tumors using multi-center patients.
- An image-based DL model was combined with a large language model to establish a diagnostic framework for ovarian tumor classification.
- Our DL model can improve the diagnosis of primary US doctors to the level of experts and might assist in surgical decision-making.

**Graphical Abstract:**

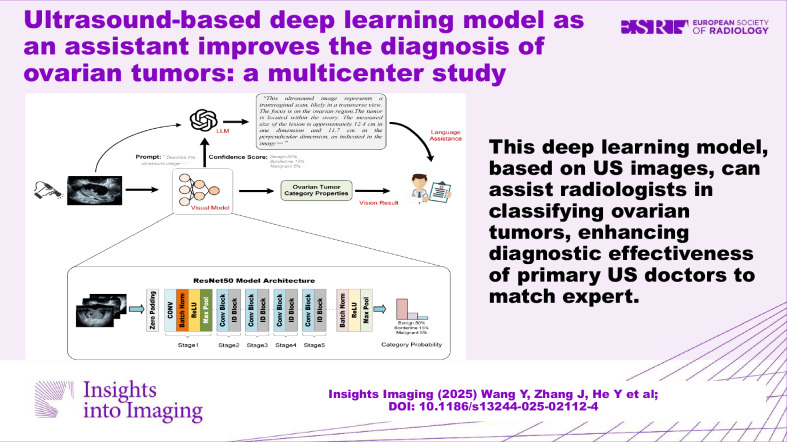

## Introduction

Ovarian tumors are the most prevalent tumors within the female reproductive system. It is typically categorized into ovarian benign tumor, borderline tumor, and malignant tumor based on whether the clinical behavior progresses to a more severe state or results in death [1]. Ovarian benign tumors are generally non-invasive, and elective surgery can be arranged [2]. Borderline ovarian tumors (BOT) describe a tumor type that falls between benign and malignant categories, which tend to grow slowly and seldom progress to malignancy [3]. It tends to occur more frequently in reproductive females, making fertility-sparing surgery a typically pursued approach [4]. Ovarian cancer (OC) is the eighth most common cancer in women, accounting for approximately 3.7% of cancer diagnoses and 4.7% of deaths in 2020 [5]. Most patients with OC are diagnosed at an advanced stage, frequently presenting with an invasion of adjacent ovarian structures. In addition, 70% of patients experience relapses post-treatment with a low survival rate. Therefore, most patients diagnosed with OC usually undergo radical surgery combined with chemotherapy [6].

US is the preferred imaging method for the early diagnosis, preoperative evaluation, and postoperative monitoring of ovarian tumors [7]. US offers the advantages of being radiation-free, convenient, and cost-effective compared to other imaging methods [8, 9]. Guidelines from the American College of Radiology indicate that the Ovarian Adnexal Reporting and Data System (O-RADS) has been widely used for US risk stratification of ovarian tumors [10]. In addition, the assessment of different neoplasias in the adnexa model (ADNEX), introduced by the International Ovarian Tumor Analysis (IOTA) in 2014, can also assist US doctors in classifying ovarian tumors [11]. However, O-RADS can only provide an approximate risk range for the tumors without categorizing them. Although some studies have suggested that microcystic patterns may be the signs of BOT, there are no widely confirmed diagnostic criteria for BOT in the US [12]. Similarly, the ADNEX model requires multiple ultrasonic features to calculate the probability. The complex evaluation system requires doctors to possess high professionalism and extensive clinical experience, as subjective factors may potentially influence the evaluation results.

Deep learning (DL) technologies hold substantial potential for medical US analysis, aiming to automate the extraction of visual features and perform pattern recognition analysis for tasks such as classification [13, 14] and segmentation [15], thereby automatically defining the attributes of images or targets. In recent years, DL models have been frequently applied in the analysis of ovarian tumors, demonstrating high accuracy in distinguishing between benign and malignant conditions [16]. However, the effectiveness of DL technologies in truly assisting clinical decision-making for ovarian tumors, especially in validating BOTs that lack human experience and standards, has received little attention. The aim of this study is to use a model that extracts features from US images of ovarian tumors, with pathological diagnosis as the learning standard, to classify benign, borderline, and malignant ovarian tumors. Furthermore, large language models (LLMs) are deep neural systems trained on massive text corpora, which capture linguistic patterns through architectures like Transformer, enabling them to comprehend, generate, and reason with natural language [17]. We used interactive text from LLMs to enhance radiologists’ decision-making capabilities regarding the nature of ovarian tumors in women by integrating visual models with LLMs.

## Materials and methods

### Study design and participants

This study was approved by the Institutional Review Board of the Second Affiliated Hospital of Fujian Medical University (IRB No. 2024323), and written informed consent was waived.

**Fig. 1:**
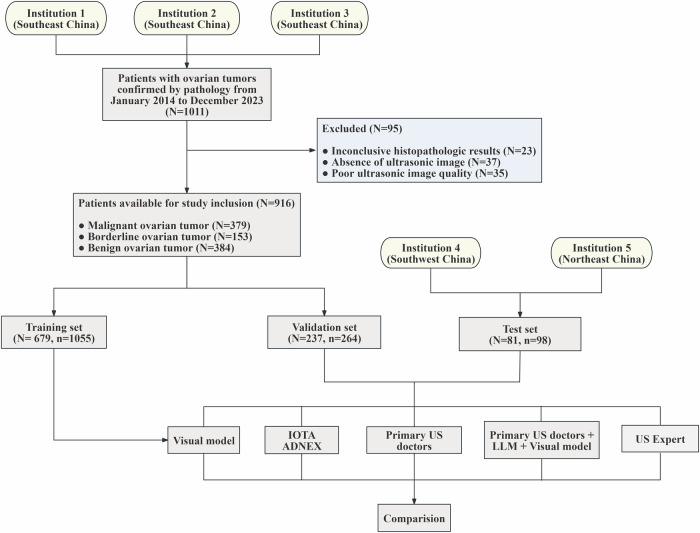
Overview of sample distribution in the study population. DL, deep learning; US, ultrasound; LLMs, large language models; IOTA, International Ovarian Tumor Analysis; ADNEX, assessment of different neoplasias in the adnexa model; *N*, number of patients; *n,* number of images

This study retrospectively enrolled patients diagnosed with ovarian tumors, confirmed by postoperative pathology and diagnosed by US at the Second Affiliated Hospital of Fujian Medical University (institution 1), Quanzhou First Hospital (institution 2), and Zhangzhou Hospital (institution 3) from January 2014 to December 2023. US images of ovarian tumors were obtained by the medical record system. The exclusion criteria included: (1) absence of images within 3 months before surgery; (2) poor quality US images; (3) unclear pathological results. Images for the test set were provided by Chengdu Third People’s Hospital (institution 4) and the First Affiliated Hospital of Dalian Medical University (institution 5). The exclusion and inclusion conditions are the same as above. Institutions 1, 2, and 3 are located in southeast China. Institution 4 is located in southwest China, and 5 is located in northeast China. Age, menopause status, preoperative cancer antigen 125 (CA125), and ascites were recorded through the clinical system. The flowchart is shown in Fig. 1.

### Ultrasonic examination

Ultrasonic inspection equipment includes GE-Voluson E8, GE-Voluson E10, Mindray Resona I9, Mindray Resona 7OB, Mindray Resona 7, Philips IU22, Philips CX-50 and Philips A70. The frequency of the abdominal probe is 3.5 to 6.5 MHZ, while the frequency of the transvaginal probe is 3 to 10 MHZ. Two-dimensional US images of ovarian tumors meeting the inclusion criteria were collected. Transverse and longitudinal images of a tumor were all included in the study. Patients with multiple ovarian tumors were included with multiple images. At least one image was selected for each side of bilateral lesions. Thus, the quantity of images exceeds the number of patients, ensuring a comprehensive inclusion of tumor images with diverse image characteristics. US images and clinical data were collected in a diagnostic system from 5 institutions and reviewed by an expert.

### Evaluation of the IOTA ADNEX model

A gynecologic US expert evaluated the US images in the validation set and test set according to the IOTA consensus [18]. The predictive variables included in the model encompassed age, serum CA125 level, type of center (tumor referral center vs. non-tumor center), maximum diameter of the lesion, maximum diameter of the solid portion, more than 10 cystic lumen (yes or no), number of papillae (0, 1, 2, 3, or > 3), sound shadow (yes or no), and ascites (yes or no). The risk of benign, borderline, stage I invasive, stage II-IV invasive, and secondary metastatic cancer was obtained after entering all predictors according to the ADNEX formulas [19]. The total malignant risk was calculated by summing the probabilities of stage I invasive, stage II-IV invasive, and secondary metastatic cancer. Relative risk was calculated by dividing the absolute predicted risk by the baseline risk, and the highest relative risk was identified as the type of ovarian tumor (benign/borderline/malignant) [20]. Subsequently, the accuracy, specificity, sensitivity, positive predictive value, and negative predictive value of the ADNEX model were calculated.

### Data for DL model

The pathology departments of each institution evaluate and classify tumors according to guidelines established by the World Health Organization and standards recommended by the International Federation of Obstetrics and Gynecology [21, 22]. The final pathological diagnosis is the reference standard for judging benign, borderline, and malignant tumors. According to the classification results, the images of ovarian tumors were categorized into three categories. A total of 1319 images from 1011 patients at three hospitals in Southeast China were used to construct the training and validation datasets. The US images are randomly split into training and validation sets in a 4:1 ratio, meaning that multiple images from the same patient could appear in both training and validation subsets. In contrast, the external test set consisted of 98 images from 81 patients at two independent hospitals in Southwest and Northeast China, and was split at the patient level, with no overlap with the training/validation institutions or subjects, enabling a comprehensive evaluation of the model’s effectiveness in assisting radiologists with decision-making. All US image labels were obtained from pathological diagnoses and standards, ensuring that the DL model would learn these US data features from golden diagnostic standards.

### Image-based model training

The visual model aims to use image processing methods to extract fine-grained features from medical images. In this study, we refer to the “visual model” as a DL model that processes US images to extract diagnostic features, in contrast to language models that interpret or generate text-based descriptions. This distinction clarifies the visual-textual roles in our DL framework. Common texture feature extractors include convolutional neural network methods and visual attention mechanisms.

To compare different image-based model architectures, we implemented three backbone networks: ResNet-50 [23] (a residual convolutional neural network), VGG-16 [24] (a plain convolutional neural network without residual connections), and ViT-B [25] (a Vision Transformer based on self-attention mechanisms). All three network architectures were initialized with weights pre-trained on ImageNet to leverage transfer learning and improve model performance. To select the best hyperparameters, parameters (learning rate in the range $[1\times {10}^{-5},1\times {10}^{-3}]$, regularization coefficients in the range $[1\times {10}^{-6},1\times {10}^{-2}]$, batch size {16, 32, 64}, and number of training epochs {50, 100, 150}.) were determined using a Bayesian inference strategy [26] during training. Visual models (ViT-b, based on attention mechanisms, and VGG-16, based on dense convolution networks) were used for comparison with the ResNet-50 [23].

To develop and validate the model, we employed a five-fold cross-validation strategy on the entire internal dataset (1319 images from three centers in Southeast China). For each fold of the cross-validation, the data was partitioned into a training set (4 folds, approximately 80% of the data) and a validation set (1-fold, approximately 20%). The performance metrics reported for the ‘validation set’ in our Results section represent the averaged results across these five validation folds to ensure robust and stable evaluation. The final model was then evaluated on the completely separate external test set. All the US images used for model training were anonymized and resized to 512 × 512 pixels. Image preprocessing included channel-wise normalization using ImageNet statistics (mean = (0.485, 0.456, 0.406); std = (0.229, 0.224, 0.225)). For training, data augmentation strategies included Gaussian blurring, random horizontal flipping, and center cropping. Validation and test images were used without augmentation. All models were trained using the categorical cross-entropy loss function, which is widely used for multi-class classification tasks. This loss measures the divergence between the predicted probability distribution and the true one-hot encoded label. It was selected for its effectiveness and stability in optimizing classification models in three-class tasks (benign, borderline, and malignant tumors). The visual models were developed on a Python 3.8 and torch 1.13 platform, using two Tesla V100 (32 G) GPUs to accelerate model training. The relevant code for the visual DL model can be accessed at https://github.com/JsongZhang/DL4OCancerUS_Radiologist.

### LLMs in US image interpretation

**Fig. 2:**
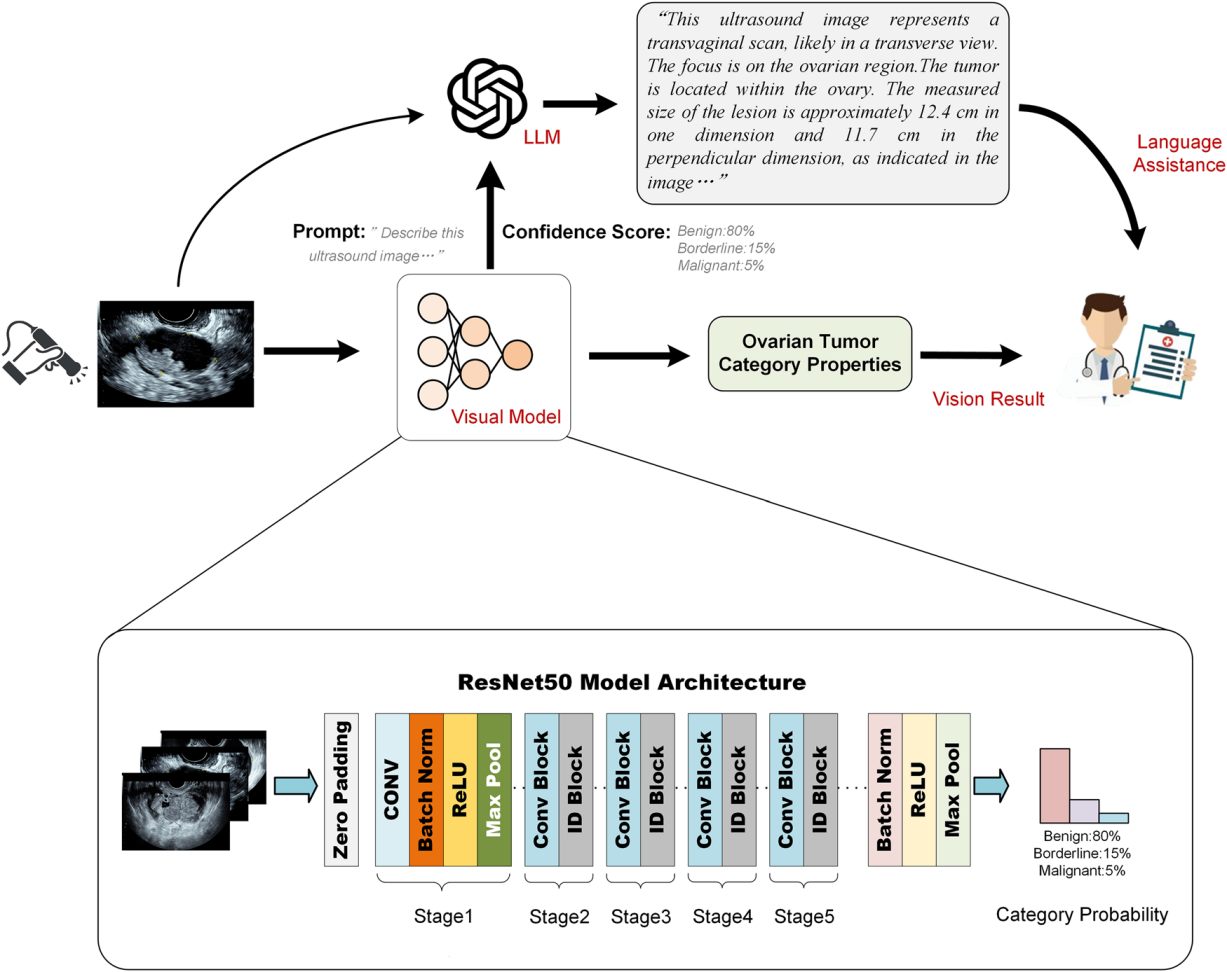
The LLMs integrating visual model confidence are used to improve radiologists’ decision-making. The probabilities for each category output by the Visual Model are fed into the LLMs as confidence scores in combination with a Prompt strategy. The prompt will guide the LLMs to generate image content information

The interactive output of the language model provides a more reliable source for assisting clinical decision-making than only visual model probability results. This study uses the aforementioned visual model’s prediction for three different types of ovarian tumors in the US as the confidence input for the language model. GPT-4o was used as the language model for text output. The Prompt can be summarized as: “*I will now give you the US images of the ovarian tumor. In clinical research, they are divided into three categories: benign, borderline, and malignant. This image is (ovarian tumor category: benign, malignant, borderline) with a confidence level of (confidence score from* visual model *precision)*…*You can use a coherent clinical description that completely removes the diagnostic conclusion. Please note that we do not provide a diagnostic conclusion in the content you provide*.” The Prompt is used to engage LLMs in generating descriptions of image properties without diagnostic [27]. Figure 2 details our workflow. During the inference phase, the visual model will predict the probabilities of different tumors, which will be passed to LLMs to generate descriptions. Radiologists can access the evaluation results of the visual model while obtaining image descriptions. The interactive DL model inference brings more credibility and interpretability to US-based diagnoses of ovarian tumors in the US. An example demonstration of a test scenario based on the proposed workflow was shown in Supplementary Fig. 1. The detailed prompt strategy for the language model can be found at: https://github.com/JsongZhang/DL4OCancerUS_Radiologist/tree/master/LLM-promt.

### Statistical analysis

SPSS 20.0 and R software (Version 3.5.2, http://www.R-project.org) were used in this study for statistical processing. Continuous variables are reported as mean ± SD and qualitative variables as frequency. The Kolmogorov–Smirnov test was used to examine the distribution of data for normality. Continuous variables were assessed by Student’s *t*-test or the Mann–Whitney *U*-test. The involved doctors in this study were five primary US doctors (Y.L.W., M.G., S.Q.C., M.Y., and P. Z., with 3–5 years of US experience) and one US expert (X.L.W.) in the medical US department. The primary US doctors were radiologists with a master’s degree in medicine who had worked continuously for more than 3 years but less than 5 years in the field. Every doctor performed three diagnostics for each sample in the Test set at 7-day intervals over a 4-week period before and after DL assistance. They identified the benign and malignant US images in the Validation set and Test set based on the O-RADS risk stratification and management system [10]. The classification results (benign/malignant) were recorded accordingly. BOTs were excluded from evaluation due to unclear ultrasonic diagnostic criteria. In addition, the tumor nature provided by the DL vision model and the image information provided by the LLMs were presented to the doctors, and the effect of the combination of visual and language modeling was verified in the same process. The high value of the US diagnosis by the primary US doctors was adopted as the final indicator of the diagnosis. Consistency calculations were used for intra- and inter-reader assessment of subject radiologists at all stages.

The Kappa consistency indicator is used to assess consistency across perspectives under a uniform standard. A confidence indicator κ greater than 0 signifies consistency, while a *κ*-value of 1 indicates a high degree of consistency.

## Results

### Baseline characteristics of patients

A total of 1417 US images collected from 997 female patients were included in this study. The images of the training set and the validation set are from institutions 1, 2 and 3, which is divided into training set and validation set according to ratio of 4:1. A total of 1055 US images were included in the training set (413 benign, 192 borderline, and 450 malignant), and 264 US images were included in the validation set (103 benign, 48 borderline, and 113 malignant) (Fig. 1). The test set of 98 images (49 benign, 21 borderline, 28 malignant) was obtained from institutions 4 and 5. The clinical and ultrasonic characteristics are shown in Tables 1 and 2. The complete histopathological classification is shown in Supplementary Table 1.

**Table 1:** Demographic and clinical characteristics of patients in the training, validation, and test sets

Characteristic	Training sets (*N* = 679)	Validation sets (*N* = 237)	Test sets (*N* = 81)	*p*
Age (year)				0.549
Mean ± SD	44 ± 15	45 ± 15	46 ± 14	
Median (IQR)	45 (33–54)	45 (33–55)	45 (37–57)	
Range	11–85	14–83	18–77	
Histology				0.032
Benign	296 (44)	88 (37)	43 (53)	
Borderline	108 (16)	45 (19)	17 (21)	
Malignant	275 (40)	104 (44)	21 (26)	
Pausimenia				0.689
Yes	251(37)	95(40)	30 (37)	
No	428 (63)	142 (60)	51 (63)	
CA125 (U/mL)				0.136
Mean ± SD	85 ± 313	73 ± 116	205 ± 658	
Median (IQR)	28 (15, 65)	32 (18, 93)	22 (12, 77)	
Range	3–5000	5–941	4–5200	
Ascites				< 0.001
Yes	217 (32)	79 (33)	1 (1)	
No	462 (68)	158 (67)	80 (99)	

There were 997 patients in the training, validation, and test sets. The analysis included measures such as mean (standard deviation), median (IQR), minimum and maximum for continuous variables, and frequencies and percentages for categorical variables. *CA-125,* cancer antigen 125

**Table 2:** Ultrasonic characteristics of patients in the training, validation, and test sets

Training set (*n* = 1055)					Validation set (*n* = 264)				Test set (*n* = 98)			
Characteristic	Benign (*n* = 413)	Borderline (*n* = 192)	Malignant (*n* = 450)	*p*	Benign (*n* = 103)	Borderline (*n* = 48)	Malignant (*n* = 113)	*p*	Benign (*n* = 49)	Borderline (*n* = 21)	Malignant (*n* = 28)	*p*
Maximal diameter of the lesion (mm)				< 0.001				< 0.001				< 0.001
Mean ± SD	64.0 ± 40.5	111.2 ± 66.5	103.8 ± 46.9		77.8 ± 35.3	130.8 ± 56.5	103.3 ± 50.6		66.0 ± 30.4	127.4 ± 49.6	99.1 ± 35.4	
Median and range	53.0 (16.0–460.0)	86.0 (16.0–290.0)	94.0 (20.0–303.0)		71.0 (24.0–204.0)	131.50 (45.0–250.0)	97.00 (23.0, 303.0)		56.0 (21.0–154.0)	116.0 (77.0–284.0)	96.5 (37.0–194.0)	
IQR	39.0–75.0	57.0–144.0	73.0–131.0		54.5–94.0	91.3–157.3	64.0–131.0		43.0–84.0	92.0–143.0	79.5–117.8	
Maximal diameter of the largest solid part (mm)				< 0.001				< 0.001				< 0.001
Mean ± SD	14.3 ± 21.8	33.0 ± 25.1	63.9 ± 26.0		18.8 ± 25.0	35.2 ± 38.4	61.0 ± 31.3		21.7 ± 27.2	34.1 ± 21.5	79.5 ± 34.7	
Median and range	0.0 (0.0–97.0)	30.0 (0.0–231.0)	62.0 (0.0–185.0)		10.0 (0.0–100.0)	30.0 (0.0–244.0)	58.0 (0.0–182.0)		9.0 (0.0–93.0)	30.00 (8.0–87.0)	78.0 (27.0–171.0)	
IQR	0.0–30.0	20.0–43.0	46.3–76.0		0.0–29.5	21.0–37.8	38.0–76.0		0.0–38.0	16.0–45.0	49.0–93.5	
Acoustic shadow	29 (7.0%)	6 (3.1%)	4 (0.9%)	< 0.001	14 (13.6%)	0 (0.0%)	1 (0.9%)	< 0.001	4 (8.2%)	0 (0.0%)	2 (7.1%)	0.47
Number of papillations				< 0.001				< 0.001				0.001
0	397 (96.2%)	109 (56.7%)	299 (66.5%)		89 (87.2%)	24 (49.9%)	86 (76.2%)		47 (95.9%)	12 (57.2%)	23 (82.1%)	
1	10 (2.4%)	24 (12.5%)	24 (5.3%)		9 (8.8%)	8 (16.7%)	6 (5.3%)		2 (4.1%)	2 (9.5%)	2 (7.1%)	
2	3 (0.7%)	27 (14.1%)	28 (6.2%)		2 (2.0%)	7 (14.6%)	4 (3.5%)		0 (0.0%)	3 (14.3%)	1 (3.6%)	
3	2 (0.5%)	10 (5.2%)	25 (5.6%)		1 (1.0%)	3 (6.3%)	4 (3.5%)		0 (0.0%)	3 (14.3%)	1 (3.6%)	
> 3	1 (0.2%)	22 (11.5%)	74 (16.4%)		1 (1.0%)	6 (12.5%)	13 (11.5%)		0 (0.0%)	1 (4.7%)	1 (3.6%)	
Cyst locules				< 0.001				< 0.001				< 0.001
0–10	411(99.5%)	169 (88.0%)	444 (98.7%)		103 (100%)	36 (75.0%)	107 (94.7%)		48 (98.0%)	12 (57.1%)	28 (100%)	
> 10	2 (0.5%)	23 (12.0%)	6 (1.3%)		0 (0%)	12 (25.0%)	6 (5.3%)		1 (2.0%)	9 (42.9%)	0 (0.0%)	

There were 1417 images in the training, validation, and test sets. Mean data are ± SD with the range in percentages. Categorical variables are shown as numbers; data in parentheses are percentages

### Intra- and inter-reader agreement

Table 3 shows the intra- and inter-reader agreement assessment between primary US doctors and US experts for the validation and test sets before using the DL model. Based on the $\kappa$ values, the agreement between internal reads was significant. Inter-reader agreement between primary US doctors (A, B, C, D, E) and a US expert (F) was substantial but fair in benign and moderate in malignant. This suggests that experience can have an impact on reading US images, which is supported not only by the imperfect agreement between primary doctors and the US expert (Table 3) but also by the varied agreement among doctors within the same experience benchmark (Supplementary Table 2).

**Table 3:** Intra- and inter-reader agreement for ultrasound diagnosis of benign and malignant ovarian tumors

	${\boldsymbol{\kappa}}$ Mean (A, B, C, D, E, F)	Agreement
­Mean in Both		
Intra (A, B, C, D, E, F)	0.746 (0.693, 0.896)	Substantial
Inter (Between Each Doctor)	0.612 (0.567, 0.761)	Substantial
­Benign		
Intra	0.622 (0.533, 0.743)	Substantial
Inter (Between Each Doctor)	0.377 (0.233, 0.458)	Fair
­Malignant		
Intra	0.629 (0.475, 0.719)	Substantial
Inter(Between Each Doctor)	0.410 (0.244, 0.443)	Moderate

Data are average *κ* statistics, with 95% CIs in parentheses. Multireader Fleiss *κ* was used for the inter-reader agreement across all readers, and Cohen *κ* was used for intrareader agreement in paired readings. A *κ* value of less than 0.21 was considered slight agreement; 0.21–0.40, fair agreement; 0.41–0.60, moderate agreement; 0.61–0.80, substantial agreement; and 0.81–1.00, excellent agreement. A, B, C, D, and E were primary US doctors, and F was a US expert.

### Diagnostic performance of DL assistance

In this study, we finally chose the ResNet-50 model (ViT-b achieved an accuracy of only 66.56%, while VGG-16 attained an accuracy of 71.15% on validation sets) (Supplementary Table 3). When evaluated using a five-fold cross-validation process, the ResNet-50 DL model achieved an average overall accuracy of 85.23% (95% CI: [80.44%, 89.00%]) across the validation sets. It achieved 89.47% [81.88%, 93.93%], 64.61% [50.44%, 76.57%], and 76.92% [68.42%, 83.79%] accuracy for benign, borderline, and malignant ovarian tumor analyses, respectively. For the test sets from Northeast and Southwest China obtained with the same US equipment, the accuracy of the model also achieved 91.80% [80.81%, 96.78%], 84.61% [65.12%, 94.42%], and 82.60% [63.96%, 93.17%] for benign, borderline, and malignant ovarian tumors, in that order. Saliency maps to show where the network focuses during classification are provided in the Supplementary Fig. 2.

### Enhance diagnostic performance by deep learning

**Fig. 3:**
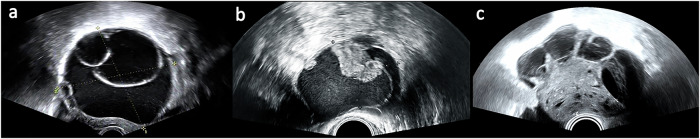
US images of benign, borderline, and malignant ovarian tumors. **a** Image of the right ovarian mass collected from a 40-year-old woman. The mass contains multiple bands and a few solid components. The diagnosis was benign by the primary US doctor and benign by the DL model. Both the primary US doctor and the DL model were diagnosed correctly. The pathological diagnosis was serous cystadenoma. **b** Image of left ovarian mass in a 45-year-old woman. There are papillae and solid components in the tumor. The diagnosis was benign by the primary US doctor and borderline by the DL model. The pathological diagnosis was borderline mucinous cystadenoma. **c** Image of left ovarian mass in a 59-year-old woman. A large number of solid components can be seen inside the tumor. The diagnosis was benign by the primary US doctor and malignant by the DL model. The DL model corrected the US doctor eventually. The pathological diagnosis was high-grade serous ovarian cancer

In the testing of radiologists, the visual model and the non-diagnostic descriptions by LLMs are used as known conditions in an offline setting. Table 4 presents the reading results of doctors A, B, C, D, E over four weeks at seven-day intervals. The DL model output was evaluated by assessing whether radiologists agreed with and adopted the model-assisted diagnosis as part of their final clinical decision. Within the validation set, primary US Doctor A’s endorsement rating improved from an initial accuracy of 80.55% for benign and malignant identification to 89.81%, B’s accuracy improved from 75.00% to 87.03%, C’s accuracy rose from 78.24% to 90.74%, D’s accuracy climbed from 77.78% to 91.20%, and E’s accuracy went up from 76.39% to 90.28%. In test sets, both A and B improved from 76.62% to 90.90%, C’s accuracy increased from 79.22% to 94.54%, D’s accuracy climbed from 76.62% to 95.95%, and E’s accuracy went up from 76.60% to 95.95%. Furthermore, the diagnostic consistency for validation and test sets for A, B, C, D, and E improved from 0.671 and 0.762, 0.412, 0.588, and 0.528, respectively, to 0.912, 0.916, 0.629, 0.701, and 0.701. DL assistance has enhanced the consistency between primary US doctors with less than five years of experience, enabling them to achieve a level comparable to US experts. Examples of enhanced diagnostic performance are illustrated in Fig. 3. Furthermore, the accuracy of the ADNEX model (validation set/test set) was 79.17%/61.22%, the Precision was 79.31%/71.69%, the recall was 79.17%/61.22%, and the specificity was 88.5%/81.21%. Compared to the ADNEX model, which includes US borderline analysis, the visual model, relying solely on US images, improved the average accuracy from 79.17% and 61.22% to 85.23% and 87.76% for validation and test sets, respectively.

**Table 4:** Performance of primary US doctors and experts with/without DL assistance

	Primary US Doctor A (validation sets, test sets) (with DL/without)	Primary US Doctor B (validation sets, test sets) (with DL/without)	Primary US Doctor C (validation sets, test sets) (with DL/without)	Primary US Doctor D (validation sets, test sets) (with DL/without)	Primary US Doctor E (validation sets, test sets) (with DL/without)	US Expert Doctor F (validation sets, test sets)
Accuracy	89.81%, 90.90%/80.55%, 76.62%	87.03%, 90.90%/75.00%, 76.62%	90.74%, 94.54%/78.24%, 79.22%	91.20%, 95.95%/77.78%, 76.62%	90.28%, 95.95%/76.39%, 76.60%	86.57%, 93.50%
Benign	86.23%, 95.83%/83.49%, 77.78%	83.49%, 93.75%/81.55%, 70.96%	87.73%, 95.83%/87.37%, 80.64%	87.38%, 95.81%/84.46%, 75.80%	86.41%, 93.75%/85.43%,70.96%	87.38%, 95.83%
Malignant	96.46%, 88.46%/69.02%, 78.94%	91.15%, 92.30%/69.91%, 78.94%	93.80%, 88.46%/69.99%, 78.88%	94.69%, 92.30%/71.68%, 77.89%	93.80%, 96.15%/68.14%, 86.84%	85.84%, 89.29%
Precision	90.45%, 89.97%/80.53%, 75.84%	87.18%, 89.76%/74.95%, 76.45%	90.93%, 94.92%/85.87%, 68.75%	91.46%, 95.96%/83.51%, 64.71%	90.51%, 95.23%/83.70%, 63.16%	86.54%, 93.32%
Benign	94.50%, 93.75%/78.50%, 87.80%	88.65%, 96.65%/73.33%, 89.74%	92.78%, 94.00% /87.38%, 79.95%	93.75%, 95.92%/84.47%, 75.51%	92.71%, 97.87%/85.44%, 71.43%	84.90%, 94.00%
Malignant	86.4%, 86.20%/82.55%, 63.88%	85.71%, 83.87%/76.57%, 63.15%	89.08%, 95.83% /85.87%, 68.75%	89.17%, 96.00%/83.51%, 64.71%	88.33%, 92.59%/83.70%, 75.51%	88.18%, 92.59%
Recall	89.53%, 90.56%/80.60%, 77.80%	86.88%, 91.32%/74.98%, 78.57%	90.59%, 93.19%/69.91%, 78.57%	91.17%, 95.12%/71.68%, 78.57%	90.11%, 95.99%/68.14%, 85.71%	86.60%, 92.60%
Benign	83.49%, 91.83%/81.55%, 73.46%	83.49%, 89.79% /74.75%, 71.42%	87.38%, 97.92% /72.58%, 86.67%	87.38%, 97.92% /73.11%, 86.05%	86.41%, 95.83%/70.97%, 89.34%	87.37%, 95.91%
Malignant	95.57%, 89.28%/79.64%, 82.14%	90.26%, 92.85%/75.22%, 85.71%	93.81%, 88.46% /69.91%, 78.57%	94.69%, 92.31%/71.68%, 78.57%	93.81%, 96.15%/68.14%, 85.71%	85.84%, 89.28%
Special	89.53%, 90.56%/80.60%, 77.80%	86.88%, 91.32% /74.98%, 78.57%	90.60%, 93.19% /87.38%, 79.59%	91.04%, 95.12%/84.47%, 75.51%	90.11%, 95.99%/85.44%, 71.43%	86.60%, 92.60%
Benign	95.57%, 89.28%/79.64%, 82.14%	90.26%, 92.85%/75.22%, 85.71%	87.38%, 97.92%/85.87%, 68.75%	87.38%, 97.92%/83.51%, 64.71%	86.41%, 95.83%/83.70%, 63.16%	85.84%, 89.28%
Malignant	83.49%, 91.83%/81.55%, 73.46%	83.49%, 89.79%/74.75%, 71.42%	93.81%, 88.46%/87.38%, 79.59%	94.69%, 92.31%/84.47%, 75.51%	93.81%, 96.15%/85.44%, 71.43%	87.37%, 95.91%
Intra $\kappa$	0.912 (0.844, 0.978)/0.671 (0.630, 0.712)	0.916 (0.849, 0.966)/0.762 (0.733, 0.791)	0.629 (0.530, 0.718)/0.412 (0.371, 0.573)	0.701 (0.622, 0.837)/0.588 (0.424, 0.638)	0.710 (0.606, 0.831)/0.528 (0.421, 0.627)	-
Benign intra $\kappa$	0.906 (0.869, 0.982)/0.679 (0.640, 0.715)	0.899 (0.840, 0.951)/0.699 (0.660, 0.735)	0.642 (0.551, 0.759)/0.400 (0.262, 0.482)	0.729 (0.733, 0.938)/0.609 (0.555, 0.752)	0.717 (0.571, 0.793)/0.538 (0.457, 0.691)	-
Malignant intra $\kappa$	0.918 (0.827, 0.951)/0.663 (0.619, 0.705)	0.933 (0.885,0.963)/0.825 (0.800, 0.847)	0.609 (0.579, 0.783)/0.423 (0.294, 0.500)	0.648 (0.518, 0.745)/0.567 (0.384, 0.599)	0.695 (0.522, 0.741)/0.517 (0.407, 0.634)	-

Data are average *κ* statistics, with 95% CIs in parentheses (BOT was not included in the comparison due to the lack of clear diagnostic criteria). A, B, C, D, E were primary US doctors, F was a US expert

## Discussion

In this study, we established a multi-center US-based DL model for the diagnosis of benign, borderline, and malignant ovarian tumors and investigated the efficacy of integrating the DL model, specifically vision and language models, into the diagnostic processes of radiologists for multi-center US ovarian tumor recognition. The findings demonstrate a significant enhancement in diagnostic accuracy among radiologists when aided by the DL model. This improvement underscores the potential of DL to enhance primary US doctors’ expertise, particularly in settings where rapid and accurate diagnoses are critical.

In the combined workflow of the visual model conclusions and language model descriptions for DL-assisted US decision-making, the study focuses on two primary US doctors who both exhibited significant improvements in diagnostic accuracy and consistency. The DL assistant successfully bridged the gap in US knowledge due to varying levels of experience, allowing primary US doctors with only three years of experience to approach the diagnostic efficacy of a US expert with ten years of experience. However, due to the lack of diagnostic guidelines for BOT, this study did not compare the performance enhancements of the DL assistant in borderline cases. Nevertheless, this workflow may contribute to the identification and development of diagnostic criteria for BOT in the future. In comparison with the multi-parametric ADNEX model, the visual analysis model based on US images achieved superior performance, suggesting the advantages of using visual model methods for analyzing US images of BOT.

Previous studies have predominantly classified BOT as malignant, distinguishing them from benign and malignant ovarian tumors [16, 28]. Chen et al [16] conducted DL modeling on US images from 304 patients with benign ovarian tumors and 118 patients with malignant ovarian tumors (including BOT). Their results showed that the sensitivity for malignant tumors was 92%, 92%, 92%, and 96% for DLdecision, DLfeature, O-RADS, and expert assessment, respectively, while the specificity was 80%, 85%, 89%, and 87%, respectively. The DL algorithm based on US images demonstrated high diagnostic performance. Similarly, Christiansen et al [28] modeled DL on 758 US images of ovarian tumors. The results showed that at a sensitivity of 96.0%, Ovry-Dx1 had a specificity similar to that of a US expert (86.7% vs. 88.0%; *p* = 1.0). Model Ovry-Dx2 had a sensitivity of 97.1% and a specificity of 93.7% when designating 12.7% of the lesions as inconclusive. DL diagnostic model accuracy is comparable to that of US experts. However, their model was unable to identify BOT independently. Given the difficulty in collecting borderline cases, we incorporated a large dataset of US images. The DL model we developed can distinguish between the three categories effectively, which is particularly meaningful for women with BOT who wish to retain their fertility function. Additionally, in contrast to our study, none of the above studies actually tested the DL model to assist US doctors in diagnosing ovarian tumors. It is a necessary experiment to evaluate the usability of the DL model. Finally, most previous studies have been single-center [16, 29–31]. Our study was conducted on a multi-center sample; thus, the generalizability and validity of the data represent a key strength. The characteristics of US images of ovarian tumors from diverse population distributions effectively demonstrate the practical applicability of the DL model in clinical US settings.

In addition, previous studies were mostly visual models and did not incorporate LLMs training [16, 31–33]. Currently, LLMs are being increasingly applied in medical diagnosis and treatment, particularly in the auxiliary diagnosis of medical images with notable progress [34, 35]. In the domain of clinical inquiry, Gungor et al [36] assessed the capabilities of LLMs, including ChatGPT-3.5, ChatGPT-4, and ChatGPT-Omni, in addressing 804 questions of varying complexity related to the diagnosis and management of gynecological cancers. The findings revealed that ChatGPT-Omni demonstrated superior performance in responding to clinical inquiries concerning gynecological cancers, underscoring its potential as a valuable decision-support tool and educational asset in clinical settings. In the domain of medical image-assisted diagnosis, Wu et al [37] evaluated three LLM chatbots-Claude-2, GPT-3.5, and GPT-4-on assigning Liver Imaging Reporting & Data System version 2018, Lung CT Screening Reporting & Data System version 2022, and O-RADS MRI, and assessed the impact of different prompting strategies. Their results demonstrated that when equipped with structured prompts and guideline PDFs, Claude-2 exhibited the potential to assign RADS categories to radiology cases based on established criteria. However, there is currently no research on integrating LLMs with DL visual models for the diagnosis of ovarian tumors. In this study, in addition to the visual model, a description of US images was generated without diagnostic constraints. Radiologists can diagnose using both the visual model-aided diagnosis and the DDL-aided diagnosis model. The interactive DL model inference brings more credibility and interpretability to actual diagnoses of ovarian tumors.

The high-performance confidence scores of the visual models combined with the descriptive text from LLMs offer US doctors a comprehensive perspective on the nature of ovarian tumors and the grading of image textures. This represents a new DL-assisted clinical paradigm. It fosters trust in DL recommendations among doctors and enhances decision-making through a multifaceted visual-linguistic model evaluation. These results support the further development and validation of multi-center, multimodal, dynamic US video analysis.

In brief, our research has the following advantages. First, it is the first to combine visual and language models to establish a DL model of assisted diagnosis for ovarian tumors based on US images. This innovative approach has been proven to enhance the diagnostic efficiency of radiologists, providing greater credibility and interpretability for the actual diagnosis of ovarian tumors. Second, a large dataset of US images from multi-center samples was utilized for the first time to develop an ultrasonic-assisted diagnostic model capable of distinguishing between benign, borderline, and malignant ovarian tumors, and the model has achieved impressive performance.

Our study also had some limitations. First, our population sample is all from Asia, and geographic homogeneity may limit the generalization of our findings to other demographic contexts. Second, our study was based on grayscale US images without blood flow images. Blood flow information from tumors will provide important insights into the vascular patterns associated with malignancy, thereby more accurately differentiating benign and malignant lesions. Third, in the diagnostic efficacy study of DL-assisted decision-making by primary US doctors, we excluded BOT due to unclear diagnostic criteria for guidelines, which caused a partial lack of comparative studies. However, the diagnostic efficiency of the DL model for BOT in this study has been proven. Fourth, a key limitation stems from the retrospective design using pre-collected static US images, which precludes real-time validation and dynamic clinical interaction analysis. Future multi-center trials with real-time data streams will address these translational constraints. Fifth, images were acquired from various US machines. Due to necessary anonymization, device-level metadata for individual images was unavailable. We employed an image-level split for the internal dataset to best simulate a real-world scenario where the model must generalize across unknown devices. Although this carries a theoretical risk of data leakage, it provides a rigorous test of generalization. Critically, the model’s generalizability was robustly validated using an external test set that was strictly patient-level disjoint and from separate institutions. We believe training on this diverse, anonymized data enhances clinical applicability, as confirmed by the strong external validation performance.

## Conclusions

The multi-center, multimodel, multisample DL assistant based on US images of ovarian tumors can provide primary US doctors with comprehensive, robust US decision-support that compensates for clinical experience.

## Supplementary information

ELECTRONIC SUPPLEMENTARY MATERIAL

## Data Availability

Data generated or analyzed during the study are available from the corresponding author upon request. The study source code can be found at https://github.com/JsongZhang/DL4OCancerUS_Radiologist.

## Abbreviations

ADNEX: Assessment of different neoplasias in the adnexa model
BOT: Borderline ovarian tumors
CA125: Cancer antigen 125
DL: Deep learning
IOTA: International Ovarian Tumor Analysis
LLMs: Large language models
OC: Ovarian cancer
RADS: Ovarian Adnexal Reporting and Data System
ResNet-50: Residual network-50
US: Ultrasound
