# Supplementary material for: Ultrasound-based deep learning model as an assistant improves the diagnosis of ovarian tumors: a multicenter study
Source: Insights Imaging. 2025 Oct 16;16:221. doi: 10.1186/s13244-025-02112-4 (PMC12532985; doi:10.1186/s13244-025-02112-4)
Supplement: Supplementary file 1 — ELECTRONIC SUPPLEMENTARY MATERIAL [file 13244_2025_2112_MOESM1_ESM.pdf]

Ultrasound-based deep learning model as an assistant improves the diagnosis of ovarian tumors: a multicenter study

ELECTRONIC SUPPLEMENTARY MATERIAL

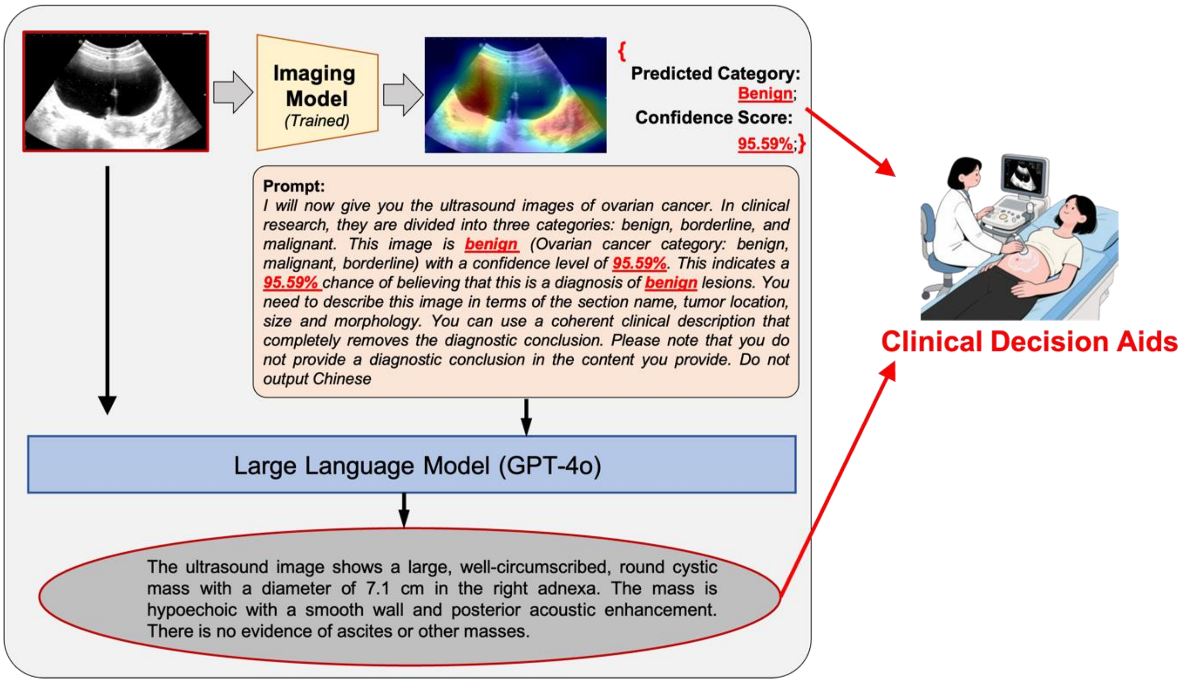

Supplementary Figure 1. An example demonstration of a test scenario based on the proposed workflow. The results of the offline inference visual model provide categorical predictions for the test image (benign, borderline, malignant), while the large language model, guided by carefully designed prompts, generates descriptive interpretations of the image content. Together, these outputs serve as decision support for clinicians. The advantage of this approach lies in avoiding full reliance on an end-to-end large model; instead, it enables clinical decision support by decoupling image content from image properties through offline training with relatively small-scale data.

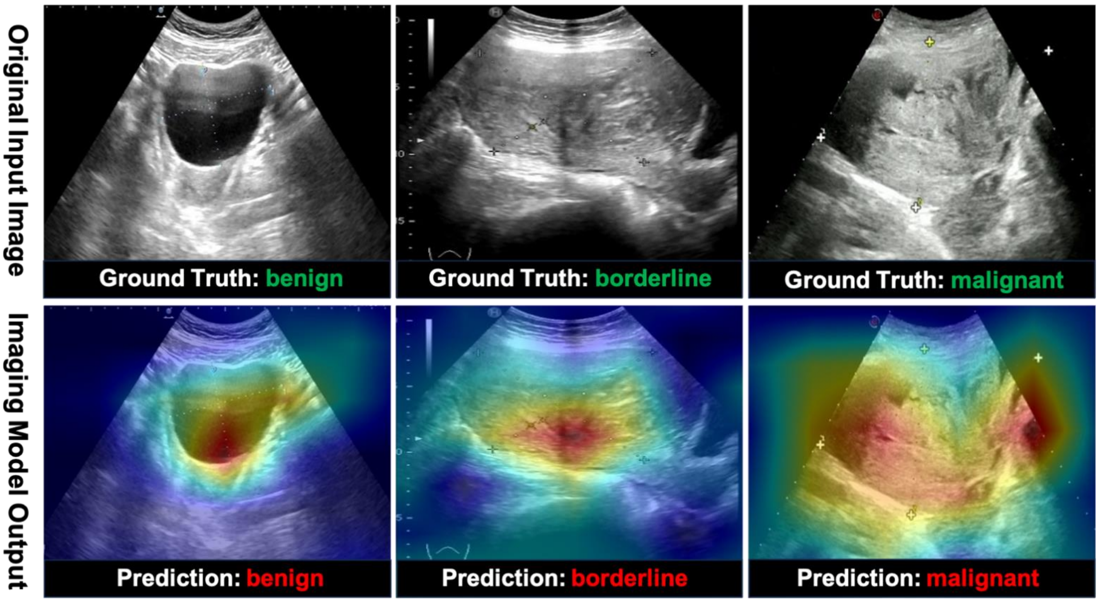

Supplementary Figure 2. Grad-CAM visualization of the trained image model. The color spectrum from red to blue indicates the transition from strong to weak model attention. The results demonstrate that the visual model attends to regions consistent with human visual focus and activates the corresponding regional features to support its decision-making.

Supplementary Table 1 Pathological categories of ovarian tumors in Training set, Verification set and Test set.

Histology	Training set (n=679)	Verification set (n=237)	Test set (n=81)
**Benign**	**296**	**88**	**43**
Simple cyst	49	20	14
Endometrioma	78	18	10
Serous cystadenoma	69	15	8
Mucinous cystadenoma	57	11	3
Mature cystic teratoma	31	21	8
Fibroma	3	1	0
Benign Brenner tumor	3	1	0
Mixed	6	1	0
**Borderline**	**108**	**45**	**17**
Mucous borderline tumor	61	24	11
Serous borderline tumor	33	15	5
Borderline Brenner tumor	5	4	1
Borderline endometrioid tumor	6	2	0
Mixed	3	0	0
**Malignant**	**275**	**104**	**21**
Serous adenocarcinoma	179	67	11
Endometrioid adenocarcinoma	20	14	3
Mucinous adenocarcinoma	21	11	2
Clear cell carcinoma	11	5	2
Malignant Brenner tumor	1	0	0
Adult granulosa cell tumor	13	3	1
Juvenile granular cell tumor	6	1	0
Carcinosarcoma	4	1	0
Immature cystic teratoma	3	0	1
Dysgerminoma	3	0	0
Metastatic ovarian tumor	9	1	1
Mixed	5	1	0

Data are presented as number.

Supplementary Table 2 Inter-reader agreement among primary radiologists (A-E)

Pair	Benign lesions ([95% CI])	Malignant lesions ([95% CI])
A vs B	0.364 [0.177, 0.537]	0.325 [0.158, 0.490]
A vs C	0.901 [0.788, 0.978]	0.983 [0.947, 1.000]
A vs D	0.840 [0.702, 0.945]	0.877 [0.788, 0.951]
A vs E	0.730 [0.578, 0.859]	0.948 [0.885, 1.000]
B vs C	0.305 [0.111, 0.486]	0.302 [0.141, 0.465]
B vs D	0.324 [0.136, 0.480]	0.256 [0.063, 0.421]
B vs E	0.514 [0.336, 0.673]	0.302 [0.128, 0.469]
C vs D	0.835 [0.695, 0.940]	0.894 [0.804, 0.966]
C vs E	0.722 [0.558, 0.860]	0.930 [0.856, 0.984]
D vs E	0.633 [0.456, 0.781]	0.858 [0.752, 0.946]

A, B, C, D, and E were primary US doctors.

Supplementary Table 3 Performance comparison of DL models on the validation set (with 95% CI).

Architecture	Class	Accuracy (%)	Precision (%)	Recall (%)	F1-score (%)
ResNet-50	Benign	89.5 [81.9, 93.9]	91.2 [84.3, 95.6]	88.7 [80.9, 93.4]	89.9 [83.0, 94.1]
	Borderline	64.6 [50.4, 76.6]	66.4 [52.1, 78.6]	61.2 [57.5, 63.0]	63.7 [59.8, 65.4]
	Malignant	76.9 [68.4, 83.8]	78.1 [69.5, 85.3]	75.3 [66.2, 82.5]	76.7 [67.5, 83.9]
VGG-16	Benign	74.8 [65.5, 82.1]	75.6 [66.0, 83.4]	72.8 [63.2, 80.7]	74.2 [64.7, 81.8]
	Borderline	59.2 [45.8, 71.5]	57.9 [45.1, 69.3]	60.2 [46.5, 72.4]	59.0 [46.1, 70.8]
	Malignant	69.5 [60.4, 77.6]	68.7 [59.5, 76.3]	70.1 [60.2, 78.0]	69.4 [59.4, 77.5]
ViT-B	Benign	71.1 [61.3, 79.5]	72.5 [62.3, 80.9]	69.3 [59.1, 77.4]	70.9 [60.5, 78.8]
	Borderline	52.2 [39.0, 65.1]	50.8 [38.0, 63.2]	53.6 [40.1, 66.2]	52.1 [39.2, 64.4]
	Malignant	59.9 [50.3, 68.9]	61.0 [50.4, 70.3]	58.2 [47.2, 67.6]	59.6 [48.5, 68.9]
